# Spray-dried lactose-leucine microparticles for pulmonary delivery of antimycobacterial nanopharmaceuticals

**DOI:** 10.1007/s13346-021-01011-7

**Published:** 2021-06-08

**Authors:** Durairaj Thiyagarajan, Benedikt Huck, Birgit Nothdurft, Marcus Koch, David Rudolph, Mark Rutschmann, Claus Feldmann, Constantin Hozsa, Marcus Furch, Karen F. W. Besecke, Robert K. Gieseler, Brigitta Loretz, Claus-Michael Lehr

**Affiliations:** 1 Helmholtz-Institute for Pharmaceutical Research Saarland (HIPS), Helmholtz Center for Infection Research (HZI), Campus E8.1, 66123 Saarbrucken, Germany; 2 Department of Pharmacy, Saarland University, 66123 Saarbrucken, Germany; 3 INM — Leibniz Institute for New Materials, Campus D2 2, 66123 Saarbrucken, Germany; 4 Institute of Inorganic Chemistry, Karlsruhe Institute of Technology (KIT), Engesserstr. 15, 76131 Karlsruhe, Germany; 5 Rodos Biotarget GmbH, Feodor-Lynen-Str. 31, 30625 Hannover, Germany; 6 Siegfried AG Hameln, 31789 Hameln, Germany; 7 Biolife Holding GmbH & Co. KG, 69126 Heidelberg, Germany; 8 Department of Medicine, University Hospital Bochum, 44892 Bochum, Germany

**Keywords:** Antibacterial nanoparticles, Benzothiazinone, Dry powder formulations, Levofloxacin, Liposomes, Respiratory infections, Tuberculosis

## Abstract

Pulmonary delivery of nanocarriers for novel antimycobacterial compounds is challenging because the aerodynamic properties of nanomaterials are sub-optimal for such purposes. Here, we report the development of dry powder formulations for nanocarriers containing benzothiazinone 043 (BTZ) or levofloxacin (LVX), respectively. The intricacy is to generate dry powder aerosols with adequate aerodynamic properties while maintaining both nanostructural integrity and compound activity until reaching the deeper lung compartments. Microparticles (MPs) were prepared using vibrating mesh spray drying with lactose and leucine as approved excipients for oral inhalation drug products. MP morphologies and sizes were measured using various biophysical techniques including determination of geometric and aerodynamic mean sizes, X-ray diffraction, and confocal and focused ion beam scanning electron microscopy. Differences in the nanocarriers’ characteristics influenced the MPs’ sizes and shapes, their aerodynamic properties, and, hence, also the fraction available for lung deposition. Spay-dried powders of a BTZ nanosuspension, BTZ-loaded silica nanoparticles (NPs), and LVX-loaded liposomes showed promising respirable fractions, in contrast to zirconyl hydrogen phosphate nanocontainers. While the colloidal stability of silica NPs was improved after spray drying, MPs encapsulating either BTZ nanosuspensions or LVX-loaded liposomes showed the highest respirable fractions and active pharmaceutical ingredient loads. Importantly, for the BTZ nanosuspension, biocompatibility and in vitro uptake by a macrophage model cell line were improved even further after spray drying.

**Supplementary Information:**

The online version contains supplementary material available at 10.1007/s13346-021-01011-7.

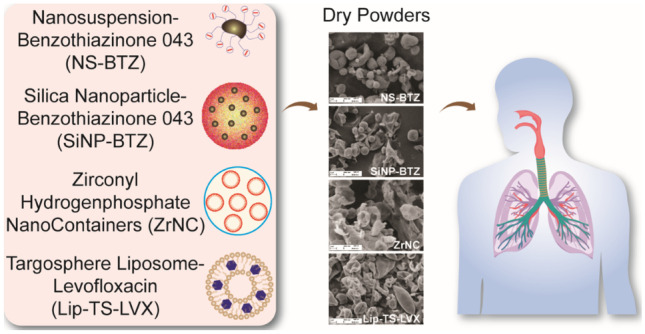

## Introduction

### Respiratory infections and multi-drug resistance

The mortality rate associated with microbial infections increases exponentially with the rate of antibiotic resistance, which calls for advanced therapeutic strategies to eradicate drug-resistant pathogens [1]. *Mycobacterium tuberculosis* (*M. tuberculosis*) is a serious healthcare issue due to the development of multi-drug resistance and difficulties in its complete eradication leading to high mortality [2–5]. Thus, the success of conventional orally administered antimicrobial therapies is limited. In contrast to systemically administered drugs, novel therapeutic strategies taking advantage of local pulmonary delivery of nanomedical formulations to the site of infection hold promise to overcome mycobacterial respiratory tract infections [2, 3].

### Anti-TB medications

Mycobacterial infections such as with *M. tuberculosis* have been a long-standing healthcare issue requiring highly potent medications [6, 7]. The revolution in drug discovery over decades brought us many lead molecules to treat such infections*.* Threateningly, bacteria continuously develop resistances by modifying internal targets of first- and second-line antimycobacterial drugs via mutating [6]. More recently, potent new antibiotics like benzothiazinone 043 (BTZ), delamanid, pretomanid, and bedaquiline were developed against *M. tuberculosis*, while some of the classical antibiotics such as levofloxacin (LVX) and rifampicin are still used in the clinic [8]. However, ensuring sufficient delivery of these antibiotics to the site of infection is critical due to the drugs’ limited solubilities and unfavorable pharmacokinetic properties [9].

Conventional oral therapy requires high antibiotic doses and a continuous treatment over several months at the cost of patient compliance and severe side effects. Converting the active pharmaceutical ingredient (API) to respirable powders administered to the lung by inhalation has opened new avenues toward the treatment of grave respiratory tract infections [10, 11]. Such formulations can be optimized for improved flow properties, within a proper size range and aerodynamic characteristics optimal for pulmonary delivery [10, 11]. After deposition in the deep lungs, however, access to the target sites of *M. tuberculosis*–infected tissues is still limited by other biological barriers. Consequently, many formulations failed to exert pharmacological effects in preclinical infection models. Cellular and non-cellular barriers encountered in the lung after deposition may hopefully be overcome by employing appropriate nanocarriers, whereas the effective delivery of nanomedicines into the lung by oral inhalation requires additional technological efforts.

### Pulmonary nanoparticle delivery

To overcome physical constrains to pulmonary delivery, nanoformulations of antimycobacterial drugs must be administered as respirable aerosols of appropriate particle diameters. Nevertheless, nebulization of nanocarriers dispersed in aqueous media may be limited by the colloidal stability of such systems [12]. API-loaded nanocarriers embedded in dry powder microparticle (MP) formulations with suitable aerodynamic properties represent an approach to overcome this obstacle. Dry powder aerosol delivery of plain antimycobacterial drugs has already been successfully demonstrated in animal models [13–15]. However, it is not trivial to generate aerosol powders of accepted pharmaceutical excipients where nanoparticle (NP) integrity and aerodynamic properties are both maintained after spray drying. Indeed, high particle aggregation was noticed in many instances, and the occurrence of crystalline structures may lead to slow release and dissolution in the alveolar lining fluid of the deeper lung [16]. Thus, it is important to verify the aerodynamic properties of the spray-dried microparticulate powders, the structural integrity of the contained nanocarriers, as well as the release and activity of the drug encapsulated in the latter.

Preserving nanocarrier integrity and minimizing physicochemical interactions that cause particle aggregation are major objectives in the development of therapeutically fit dry powder preparations [17]. Dry powders can be prepared from suspensions of nanoparticles by either spray drying or freeze drying. Freeze drying may result in cake formation, cracking, and disruption of the nanosystem [18], which sometimes even causes leakage of the cargo. In addition, it may not be possible to define the size and shape of the MP formulation of freeze-dried preparations [19]. Alternatively, spray drying allows to generate particles of defined size and shape, with appropriate aerodynamic properties for their aerosolized delivery to the lung [12, 20]. However, the physicochemical properties and concentration of both, excipient(s) and NPs, have been demonstrated to affect the size and aerodynamic properties of the MPs [21].

We here developed lactose-leucine–based MPs loaded with various NPs and studied their principal suitability for lung administration. According to the literature, leucine and isoleucine can be used to keep the surface wetted since these molecules tend to stay in the interface when mixing with lactose solution, thus forming a coated surface after spray drying [22–24]. We found that the formation of MPs by spray drying of lactose was indeed improved by employing the hydrophobic amino acid, leucine. As a result, particle aggregation was prevented, and particle integrity was maintained. The MPs produced by encapsulating various kinds of NPs showed different morphologies, which may result from a variation in droplet formation with the variation in NP load. Previous reports on spray drying and particle formation clearly demonstrated the various stages in particle formation from the droplets during the spray-drying process [25].

This study is part of the governmentally funded multi-disciplinary project “Antibiotic Nanocarriers for Therapeutic Inhalation against Tuberculosis” (ANTI-TB). In order to address the obstacles sketched above, both organic (liposomal) and inorganic nanocarriers (silica-NPs and zirconyl hydrogen phosphate containers) loaded with LVX or BTZ, respectively, as well as some excipient-low nanosuspensions were developed by partners of this consortium. These nanoparticles were selected after the rigorous optimization of our partners with various nanosystems, antitubercular agents, drug load, and stability. Based on the investigation by the consortium partners, the selected best candidate in each category was taken for further studies. Indeed, the consortium planned to have organic formulations (liposomal formulations from Rodos Bio Target) and inorganic nanocontainers (various metal oxide particles from KIT) and the simplest API nanosuspension; thus, we have selected these four different systems for this work. Based on these types of nanopharmaceuticals, our study focuses on the preparation of lactose-/leucine-based MPs with favorable aerodynamic properties. Besides, all dry powder formulations were characterized in terms of MP morphology, NP integrity, and colloidal stability as well as API crystallinity. Based on such data, the most promising candidate was further investigated for biocompatibility and cellular uptake by the human monocytic THP-1 leukemia cell line [26].

## Materials and methods

### Materials

Lactose, leucine, fluorescein sodium, and LVX were obtained from Sigma-Aldrich, Taufkirchen, Germany. 1-octanol was purchased from Honeywell (Fisher Scientific, Schwerte, Germany). The BTZ nanopharmaceuticals (NS-BTZ and SiNP-BTZ) as well as zirconyl hydrogen phosphate nanocontainers (ZrNC) [27] were synthesized by the Feldman lab at the KIT (Karlsruhe, Germany). According to scanning electron microscopy and statistical evaluation, NS-BTZ and SiNP-BTZ NPs exhibited mean diameters and size distributions of 60 ± 26 nm or 53 ± 17 nm, respectively. In nanocontainers, BTZ had been encapsulated by a zirconyl hydrogen phosphate or a silica shell to disperse the lipophilic drug in water [27]. The ZrNC employed in this present study were not loaded with BTZ. Finally, LVX-loaded liposomes based on the patented TargoSphere® technology [28] (Lip-TS-LVX) were formulated by Rodos Biotarget GmbH (Hannover, Germany). The human monocytic THP-1 leukemia cell line (ACC 16) was obtained from the German Collection of Microorganisms and Cell Cultures GmbH (DSMZ; Braunschweig, Germany).

### MP formulation

Lactose solutions of 2.5% w/v were prepared by dissolving the lactose in Milli-Q water overnight using a stirrer at 650 rpm. Further, 1 g of leucine was added to volumes of 100 mL of the lactose solution and stirred at the same speed until dissolved completely. Such solutions were sterile-filtered (0.45 µm) and stored in sterile containers. To prepare dry powder formulations of various NPs, NP suspensions were added to the above solutions, along with fluorescein sodium solution, and gently dispersed for 5 min. For the respective amounts and concentrations used for MP preparation, see Table 1. Using a Büchi-90 nano spray dryer (Flawil, Switzerland), the whole contents were spray-dried under the following conditions (gas flow 112 L/min, frequency 122 kHz, inlet temperature 87 °C, outlet temperature 35 °C, pump 30%, spray 80%, pressure 37–38 mbar, and room humidity 20–30%). MPs thus gained were collected using a plastic scrapper, transferred to glass containers wrapped with aluminum foil to avoid photo bleaching, and stored in a desiccator at RT. For the optimization, initially, 2.5% and 7.5% lactose particles were produced and characterized. Further, its aerodynamic properties have been increased after the addition of 1% leucine in the formulation.

**Table 1:** Sample designations of NPs and MPs, as well as NP and API contents per 15 mL of spray-drying dispersion. The yield of dry powder formulations was determined by weighting the powder and calculated as percentages of the solid content. The highest API contents were achieved with BTZ-NS-MPs as a combination of both a high API concentration in the nanocarrier and a high particle concentration had been provided in the stock solution. The nanospray dryer uses an electrode for charge-based particle collection. Observed yields in the range of ~ 75–80% are plausible, since the complete collection is impossible due to technical reasons

NP names and abbreviations	MP name	NP stock solution added [μL]	NP load [mg/g of powder]	API load measured/(theoretical) [mg/g of powder]	MP yield (%)
–	LL-MPs (Lactose-leucine microparticles)	0	0	0	81
NS-BTZ (nanosuspension-benzothiazinone)	NS-BTZ MPs	375	8.250	2.053/(2.983)	78
SiNP-BTZ (silica nanoparticle-benzothiazinone)	SiNP-BTZ MPs	1203	8.632	0.062/(2.718)	80
ZrNC (zirconyl hydrogen phosphate nanocontainers)	ZrNC MPs	150	-	0/0	76
Lip-TS-LVX (TargoSphere liposome-levofloxacin)	Lip-TS-LVX MPs	150	1.019	0.724/(1.019)	83

### Characterization of dry powders

Scanning electron microscopy: Spray-dried MPs were deposited gently on a carbon tape (mounted on metal stage) using a spatula, and a mild airflow was applied to remove loosely bound excess particles from the surface. Samples were then gold-coated (Quorum Q150R ES) and examined in a field emission scanning electron microscope (Zeiss EVO MA15 LaB_6_; Jena, Germany) at 5.0 kV and × 20,000 magnification.

Static light scattering: To measure the particles’ diameters via static light scattering, 20 mL of 1-octanol in a quartz cuvette was used as a blank in the Horiba Partica LA-960 Laser Scattering Particle Size Distribution Analyzer (Darmstadt, Germany). The powders were dispersed in 1-mL octanol and sonicated for 1 min to achieve proper dispersion. Further, this suspension was gradually added to the cuvette containing octanol and mixed by magnetic stirring at maximum speed. The particle size distribution was calculated as based on the reduction in transparency and scattering values. Median values of each sample were plotted.

FT-IR spectroscopy: Powder samples were placed on the crystal of the Vertex 70 Frontier Optical FT-IR spectrometer (PerkinElmer, Hamburg, Germany), and the respective transmittances were analyzed from 4000 to 650 wave numbers. As controls, samples of the non-encapsulated APIs were analyzed accordingly.

X-ray diffraction: Compact, thin pellets of all dry powder formulations (including lactose-leucine physical mixture), BTZ, and LVX were prepared using sample holders and subsequently analyzed with an X-ray diffractometer (XʼPert Pro MPD, Almelo, Malvern Panalytical, The Netherlands). In addition, the NP control in water was analyzed to verify the NP-related background.

### Aerodynamic properties of the dry powder formulations

The aerodynamic properties of the dry powder formulations were measured using the COPLEY Next Generation Impactor (NGI) (Colwick, UK) connected with an Akita air flow generator (Bremen, Germany). Ten milligrams of each powder sample was weighted and filled in clear gelatin capsules. The NGI plates were coated with polyalkylene glycol ether (Brij®35) + glycerol coating solution to ensure proper particle binding on the surface of the plate upon air circulation. Capsules were placed into a HandiHaler® (Boehringer Ingelheim, Ingelheim, Germany) and pierced once to break the capsule. The inhaler was placed onto the rubber mouth-shaped adaptor before applying pressurized air (suction) for 4 s at a flow rate of 60 L/min. Samples deposited on all 8 stages, pre-separator, tube, and empty capsule were collected using Milli-Q water. Collected samples were analyzed at λ_EX_ = 460 nm and λ_EM_ = 515 nm using a TECAN 96 well plate reader (Männedorf, Switzerland) for determining the amount of MP-encapsulated fluorescein. The fine particle fraction collected in stages 1–5 was considered as the respirable fraction based on their size range (i.e., 1–5 μm). Exhaled powder fractions were calculated by subtracting the cumulative deposited particle amount in all stages, tube, pre-separator, and capsule from 100%. Results from independent experiments are depicted as means ± standard deviation (SD).

### Stability of the nanosystem in spray-dried powders

Confocal microscopy: Amounts of 1 mg of powder were admixed with 50 µL of 1-octanol and sonicated for 1 min for dispersion. Of these samples, aliquots of 1 μL were placed onto the microscopic slide and sealed with coverslips. Images were taken with a Leica DMi8 Confocal Microscope (Leica, Mannheim, Germany) equipped with a × 63 water immersion objective (HC APO CS2 63 × /1.20) (Leica, Mannheim, Germany), and image analysis was performed with LAS X software (Leica Application Suite X). The MPs were visualized by the green fluorescence of fluorescein, and the NPs were labeled with lumogen red in case of the inorganic NCs and PE-Texas red in case of the organic NCs to determine the NP distribution within the MPs.

FIB-SEM: Samples were analyzed as previously described [29], with slight modifications. Briefly, powders were dispersed in ethyl acetate and dried on a 5-mm × 5-mm silicon wafer under ambient conditions before the samples were glued on Al stubs using silver paste. An FEI Versa 3D FIB (Thermo Fischer, Dreieich, Germany) was used in high-vacuum conditions to select an area (approx. 30 µm × 4 µm). Cross sections were prepared after deposition of ~ 2 µm Pt before using the Ga ion beam at 30 kV accelerating voltage and a 7-nA ion current. Finally, the samples were polished at 30 kV/1 nA Ga ion beam. Cross sections of liposomes were visualized using secondary electron imaging (ETD detector) at 5 kV accelerating voltage. SEM imaging of the cross sections was also performed using an FEI Quanta 400 FEG (Thermo Fischer, Dreieich, Germany) under high-vacuum conditions. Secondary (ETD detector) and back-scattered (SSD detector) electron images were acquired under 52° tilting angle at 10 kV accelerating voltage.

Size and zeta potential: NP size and zeta potential were measured using Zetasizer Nano (Malvern Panalytical, Grovewood, UK). The spray-dried powders were dissolved in Milli-Q water, and the measurements were compared to the original values of the non-spray-dried nanomaterials. All samples were diluted for an attenuation of around 10^6^–10^9^. Three independent measurements were performed per run, and the SD was calculated as based on these values.

### Active pharmaceutical ingredient quantification

APIs were quantified using a Dionex Ultimate 3000 U-HPLC (Thermo Fischer, Dreieich) equipped with a Synchronis C18 50 × 2.1 mm, 1.7 µm column (Thermo Fischer, Germany) and a UV–VIS detector (Thermo Fischer, Germany). (i) In case of LVX, 10 mg of dry powder was dissolved in 1 mL PBS containing 0.1% Triton-X and eluted with a 18% of mobile phase A (acetonitrile (ACN)) and 72% of mobile phase B (0.5% trimethylamine buffer at pH 2.5) at a flow of 0.3 mL/min (see Supporting Information, Fig. 7). (ii) In case of BTZ043 (Selleckchem; Houston, TX, USA), powder was dissolved in a 60:40 mixture of ACN:PBS at a concentration of 10 mg/mL of dry powder. A binary solvent gradient was applied at a flow rate of 0.3 mL/min and programmed as follows: 80% H_2_O and 20% ACN at 0 min to 0.5 min, progressing linearly at 5% H_2_O and 95% ACN at 2 min, followed by the hold in H_2_O 5% ACN to 95% for 1 min, and finally returning to the initial gradient until 5 min (*cf.* Supporting Information, Figs. 8 and 9). Data analysis was performed with Chromeleon 7 software (Thermo Fischer, Germany).

### Cytotoxicity of NS-BTZ

THP-1 cells were cultured in RPMI 1640 supplemented with 10% FCS during passages 16–21 and seeded in 96-well plates (Greiner; Fisher Scientific, Germany) at 1 × 10^5^ cells/200 µL containing 25 ng/mL phorbol 12-myristate 13-acetate (PMA), which initiates macrophage differentiation [30]. Seventy-two hours post seeding, the cells were incubated with Hank’s balanced salt solution (HBSS) containing 84 μg/mL, 42 μg/mL, or 8.4 μg/mL of dissolved NS-BTZ particles for 4 h at 37° and 5% CO_2_. Following incubation, the cells were washed twice with PBS and incubated for another 4 h with 0.5 mg/mL 3-[4, 5-dimenthylthiazol-2-yl]-2,5-diphenyl tetrazolium bromide (MTT) (Sigma) in HBSS. Subsequently, the supernatant was removed, and formazan crystals were dissolved in 200 µL dimethyl sulfoxide (DMSO) (Sigma-Aldrich). The absorbance was read at 550 nm with a Tecan Infinite M200Pro plate reader (Männedorf, Switzerland). As a control for 100% viability, the cells were incubated in HBSS only, whereas 1% Triton-X-treated cells served as a negative control (i.e., 0% viability). HBSS blanks were subtracted from the positive and negative controls, and viability percentages were calculated as

$$\frac{{A}_{sample}-{A}_{blank}}{{A}_{neg Control}-{A}_{blank}}\times 100$$

### Cellular uptake

THP-1 cells were cultured as described above and seeded at 1 × 10^4^ cells/well/0.5 mL in 8-well chamber slides (Greiner Bio-One; Kremsmünster, Austria) containing 25 mg/mL PMA. After 72 h, the cells were incubated for 2 h, which dissolved either NP or MP samples at a final BTZ043 concentration of 42 µg/mL in the respective formulation. Treated cells were then washed twice with PBS and fixed with 4% paraformaldehyde (PFA) in PBS for 10 min at RT. For actin staining, cells were incubated for 30 min with 100 µL of 1:1000 Alexa Fluor 488-Phalloidin stock solution (Thermo Fisher Scientific, Waltham, MA, USA) after permeabilization and were blocked for 20 min with 1% w/v BSA and 0.1% w/v saponin in PBS. Nuclei were counterstained with 200 µL of a 1-µg/mL 4′,6-diamidino-2-phenylindole (DAPI) solution (Sigma-Aldrich) for 15 min. Images were taken with a Leica DMi8 Confocal Microscope equipped with a × 63 water immersion objective (HC APO CS2 63 × /1.20), and image analysis was performed with LAS X software (Leica Application Suite X).

## Results

### Production of spray-dried MPs

**Fig. 1:**
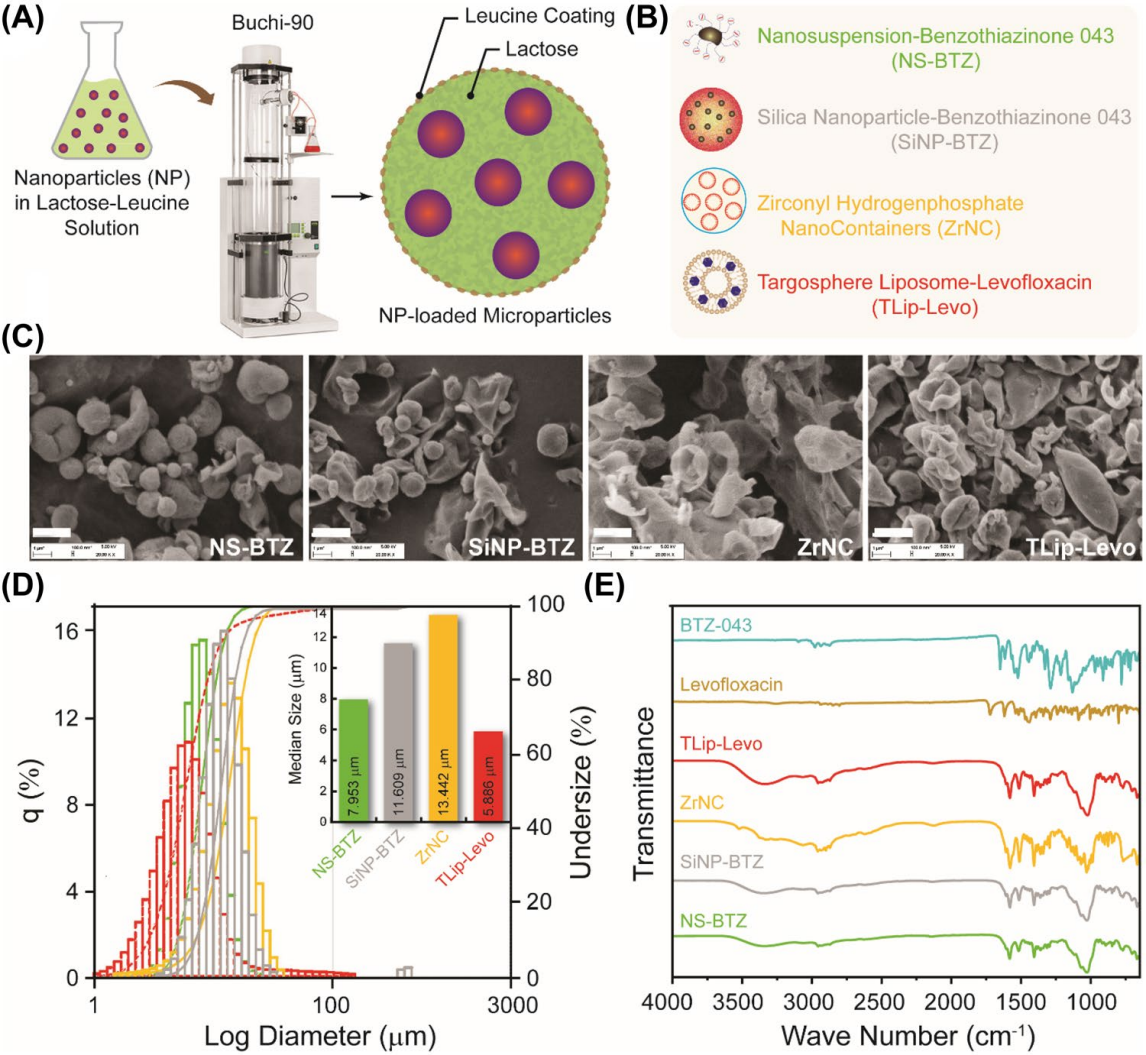
(**A**) Schematic representation on the production of MPs from NPs suspended in 2.5% lactose + 1% leucine solution. (**B**) NPs used in this study and their description. (**C**) Scanning electron micrographs of MPs (scale bar 2 µm). Characterization of MPs by static light scattering (**D**) and Fourier-transform infrared spectroscopy (**E**)

Based on preliminary spray-drying experiments (data not shown), the concentrations of lactose (2.5% w/v) and leucine (1.0% w/v) were considered as suitable and fixed. Table 1 summarizes MP designations as well as NP and API contents, and the obtained yields of each MP formulation. Figure 1a and b depict the schematic workflow and structure of the different nanopharmaceuticals to be incorporated in MPs. The concentration of fluorescein as a marker compound was kept constant in all the formulations, while the types and amounts of incorporated NPs were changed. Different physicochemical properties of the resulting MPs are therefore attributed to the respective nanomaterials used for spray drying.

The incorporation of NPs into the lactose/leucine MPs changed the MP’s morphological properties. Plain lactose-leucine MPs (LL-MP) were round in shape (*cf.* Supporting information, Fig. 5), having a rough surface. The final concentrations of lactose (2.5%) and leucine (1%) have been optimized based on results that indicate that the leucine improves the aerodynamic properties and that the lower lactose concentration would be advantageous to achieve higher drug load. (*cf.* Supporting information, Fig. 5). As shown in Fig. 1c, the shape of the LL-MPs was not changed considerably by incorporating NS-BTZ, whereas SiNP-BTZ displayed a dramatically changed morphology with only few particles retaining a round shape. LL-MPs containing ZrNC were aggregated, whereas the Lip-TS-LVX formulation was changed more profoundly, with the occurrence of elongated structures. Morphological changes were also reflected in the MPs’ median geometric size as analyzed by static light scattering (Fig. 1d).

Although having the smallest median size, Lip-TS-LVX-MPs showed more tailing of undersize particles compared to the three other MPs. However, the median size of the Lip-TS-LVX-MPs was not increased drastically in contrast to the other MPs showing increment in the median size compared to the plain LL-MPs. In general, with increasing structural complexity of the nanosystem, the median size of the MPs was increased. In the NS-BTZ system in which the API was solubilized and stabilized by sodium dodecyl sulfate (SDS), thus representing the “simplest” nanosystem, only a mild increment in particle size was observed. In contrast, the size of the SiNP-BTZ particles was increased evidently, which corresponds to the structures visible by SEM (Fig. 1c). Similarly, ZrNCs also showed a drastic increase in median particle size, which likewise corresponded to the SEM images.

Further characterization by FT-IR revealed for all the dry powders some peaks around 3500–3250/cm, indicative of the presence of water molecules (Fig. 1e). Peaks of LVX or BTZ, respectively, were found merged with those of lactose-leucine in the corresponding dry powders. All powder formulations showed complex FT-IR patterns due to the presence of the NPs compared to the non-encapsulated APIs. All MP formulations were analyzed further for their API contents. The presence of API was measured by HPLC, and the loading of the selected formulations was calculated (Table 1). The BTZ in the nanosuspension was detected before and after spray drying (see Supporting information, Fig. 8), and the percentages of drug recovery calculated from these values were ~ 70% similar to the recovery of LVX from Lip-TS-LVX. In contrast, the BTZ content detectable in the SiNP-BTZ was very low (≤ 2%). However, this had already been observed for the same MPs before spray drying and may be due to a poor release from their dense, hydrophilic silica shell.

### MP integrity after spray drying

**Fig. 2:**
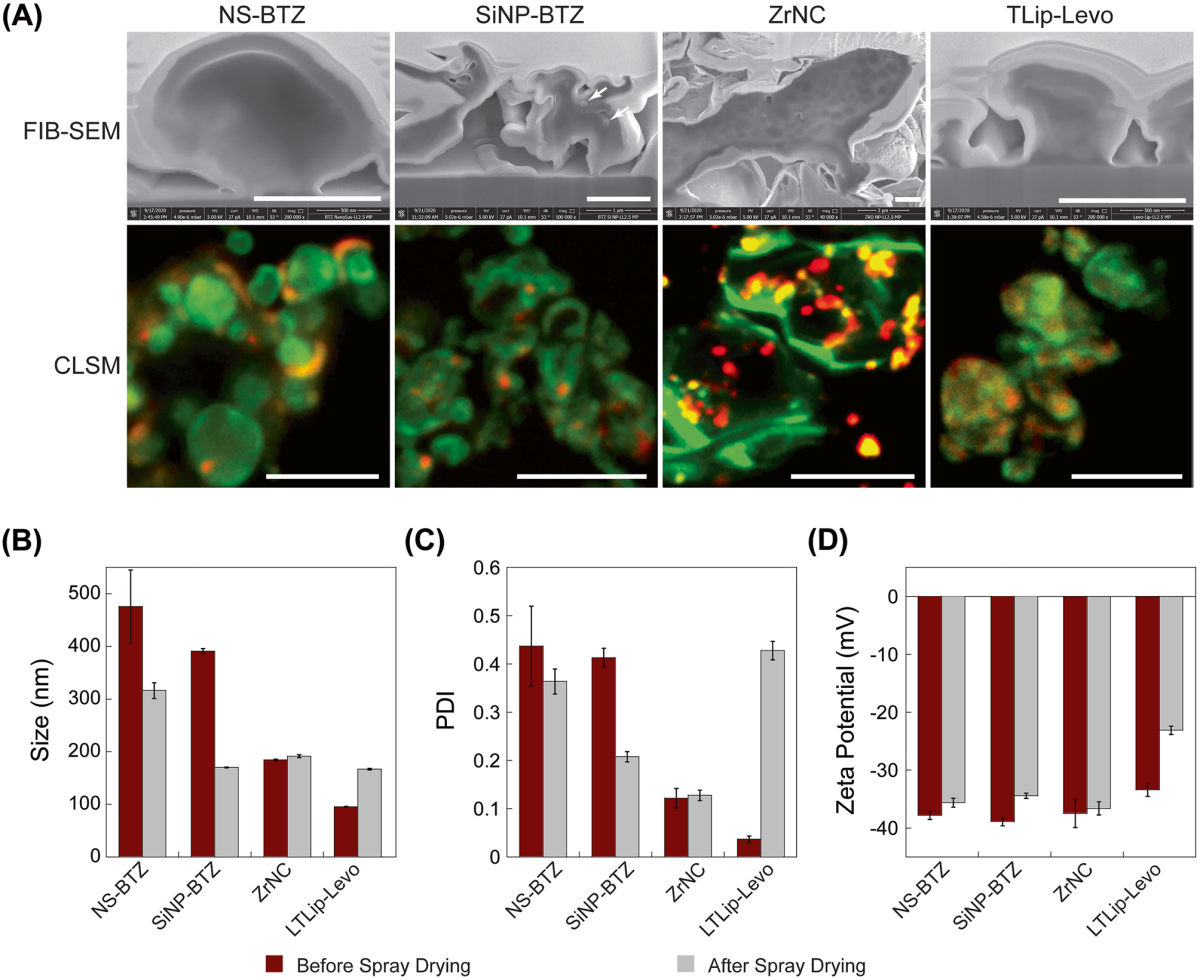
(**A**) Characterization of various MPs by focused ion beam scanning electron microscopy (arrows point at the NPs inside the MPs; scale bar 1 µm) and confocal microscopy (scale bar 2 µm). Colloidal properties of the NPs before and after spray drying by measuring their size (**B**), PDI values (**C**), and zeta potential (**D**)

While spray-dried MPs attained various shapes and sizes depending on the NP loads, the internal structural arrangements of the NPs were investigated by FIB-SEM (Fig. 2a). Powder particles were cut to allow a view on internal structures. The plain and dense interior of the NS-BTZ was similar to that of the cargo-free plain LL-MP particles (Fig. 2a). SiNP-BTZ MPs were contracted and displayed some NP structures inside the MPs. ZrNCs were highly aggregated, with some hollow meshes visible on the inner surface. The structure of Lip-TS-LVX-MPs was also found shrunk and hollow (Fig. 2a).

Confocal microscopy of the various powder formulations (Fig. 2a) provided further information about the NP distribution within the MPs. The green fluorescence signal from the uniformly dispersed fluorescein allows to identify structures of the MPs. Red fluorescence was due to Texas red within the NPs, and all nanocarriers revealed their location in the MPs. For NS-BTZ representing the smallest and least defined nanostructures among all MP cargos, the particles did not become visible as distinct shapes, thus obviously falling below the limit of microscopic resolution (Fig. 2a). In contrast, the solid SiNP-BTZ were clearly visible and were well-dispersed inside the MPs (Fig. 2a). The Lip-TS-LVX showed some aggregation tendency (Fig. 2a), and aggregation was even more pronounced in case of the ZrNCs (Fig. 2a).

### NP properties after releasing from MPs

We determined the colloidal properties of the NPs after being released from the MPs upon their dispersion in an aqueous medium. The average size of both BTZ NPs was reduced after spray drying, whereas the liposomal size was increased due to aggregation in the dissolved MP samples (Fig. 2b). The average size of ZrNC remained stable before and after spray drying. Indeed, the PDI values showed the same trend, which was found particularly pronounced for the spray-dried Lip-TS-LVX. The zeta potential of all nanocarriers was moderately increased after release from the spray dried MPs, with the most pronounced change measured for the Lip-TS-LVX. Although the colloidal properties follow a similar trend in the presence of 0.1 mg/mL simulated lung fluid (SLF), following the recipe described earlier [31] (*cf.* Supporting information, Fig. 6), there was slight increment in size and zeta potential which confirms the SLF components did have very slight influence on the nanoparticles physiochemical properties in this case.

### Aerodynamic properties of the MPs

**Fig. 3:**
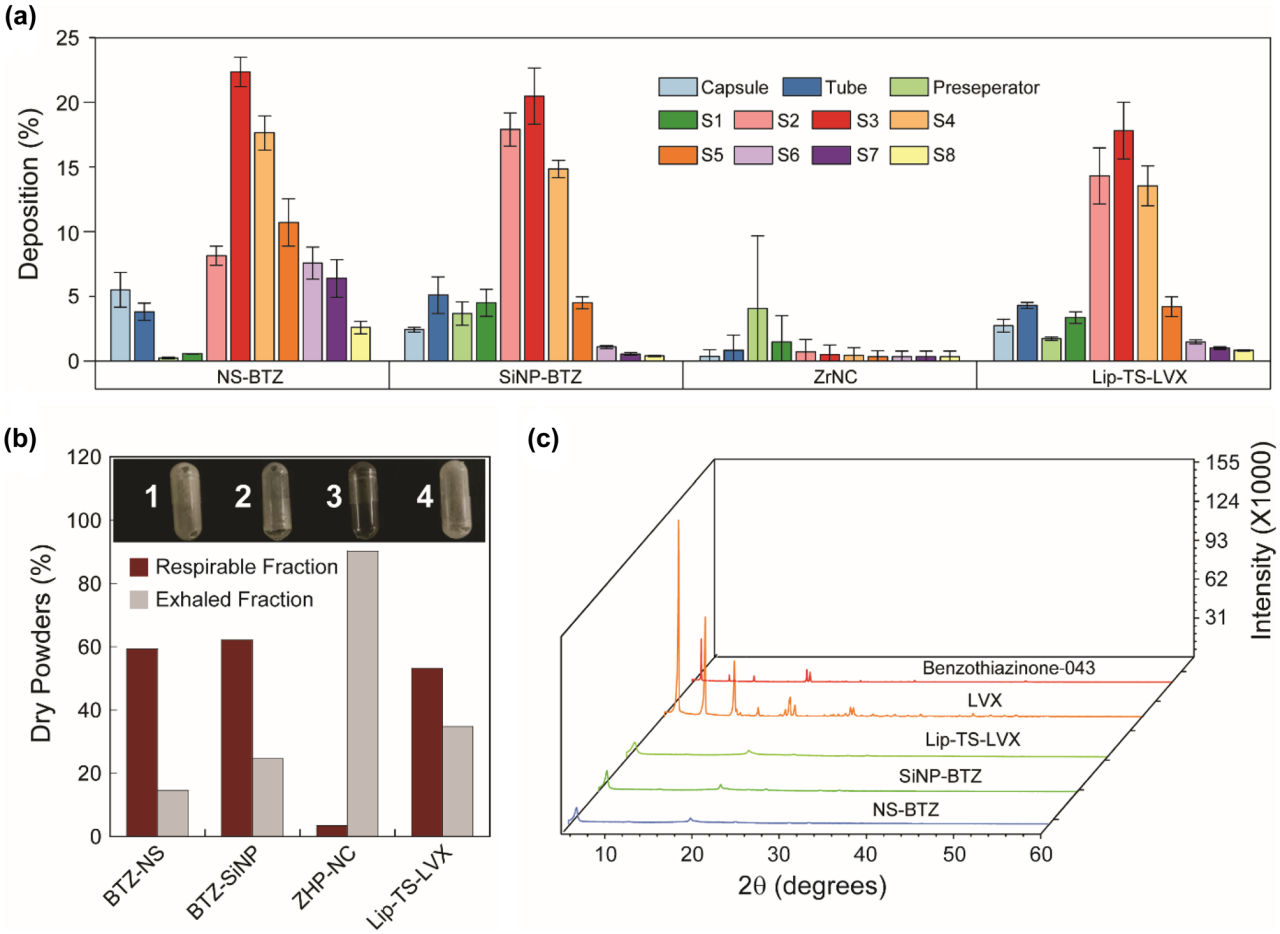
(**a**) Aerodynamic properties of various MPs studied by next-generation impactor. (**b**) Analysis of respirable fraction calculated from fine particle fraction (FPF) (insert: empty capsules after completion of the NGI experiments demonstrating the variation in stickiness among the formulations. (**c**) Powder XRD measurements of the dry powder samples

Spray-dried powders were analyzed using a next-generation impactor (NGI) for quantification of respirable particle fractions [32]. All spray-dried products showed distinct aerodynamic properties based on their sizes, shapes, and nature of NP cargos as shown in Fig. 3a. The NS-BTZ MPs possessed good aerodynamic properties as the powder particles were mostly deposited in stage 2, with moderate amounts also recovered in stages 3–5. The amounts deposited in stages 2–5 reflect the fraction available for deep lung deposition. Higher amounts of the SiNP-BTZ powders were deposited in stage 2 — yet less in fractions 5–8, which represent the exhaled portion. The depositions measured for Lip-TS-LVX and SiNP-BTZ were almost identical (Fig. 3a). Surprisingly, ZrNCs did not deposit in any of the stages, as they were lighter due to their high bulk volume and low density, which correlates with structures visible upon FIB-SEM analysis (Fig. 2a). In order to compare the particle fraction available for deep lung deposition, the cumulative deposition percentage from stages 1–5 was calculated and defined as respirable fraction (fine particle mass — FPM) [32] (Fig. 3b), while the material that crossed stage 8 was considered as exhaled fraction. The respirable fraction was thus almost 60% in both BTZ formulations, and the exhaled fraction ranged between 15 and 20%. In case of the Lip-TS-LVX, the respirable fraction was slightly reduced, and the exhaled fraction was increased. Figure 3b shows the representative capsules after the experiment where the stickiness of the powders to the capsule walls corresponded to their hygroscopicity. The slightly stickier powder of the Lip-TS-LVX showed a reduced respirable fraction, while the non-adherent ZrNC-MPs were not recovered in any of the stages. Indeed, leucine played a critical role in the aerodynamic properties of the MPs and the absence of leucine severely affected the aerodynamic properties of the MPs (*cf*. Supporting information, Fig. 5).

Finally, dry powders with promising respirable properties were checked for their crystallinity by powder X-ray diffraction measurement. The NP-free control MPs were amorphous (Supplementary information, Fig. 5g); this did not change with the addition of all three API-containing nanomaterials comprising NS-BTZ, SiNP-BTZ, and Lip-TS-LVX (Fig. 3c). The NPs in solution did not show any crystalline structure either (*cf.* Supporting information, Fig. 5).

### Cytotoxicity and uptake studies using THP-1 cells

**Fig. 4:**
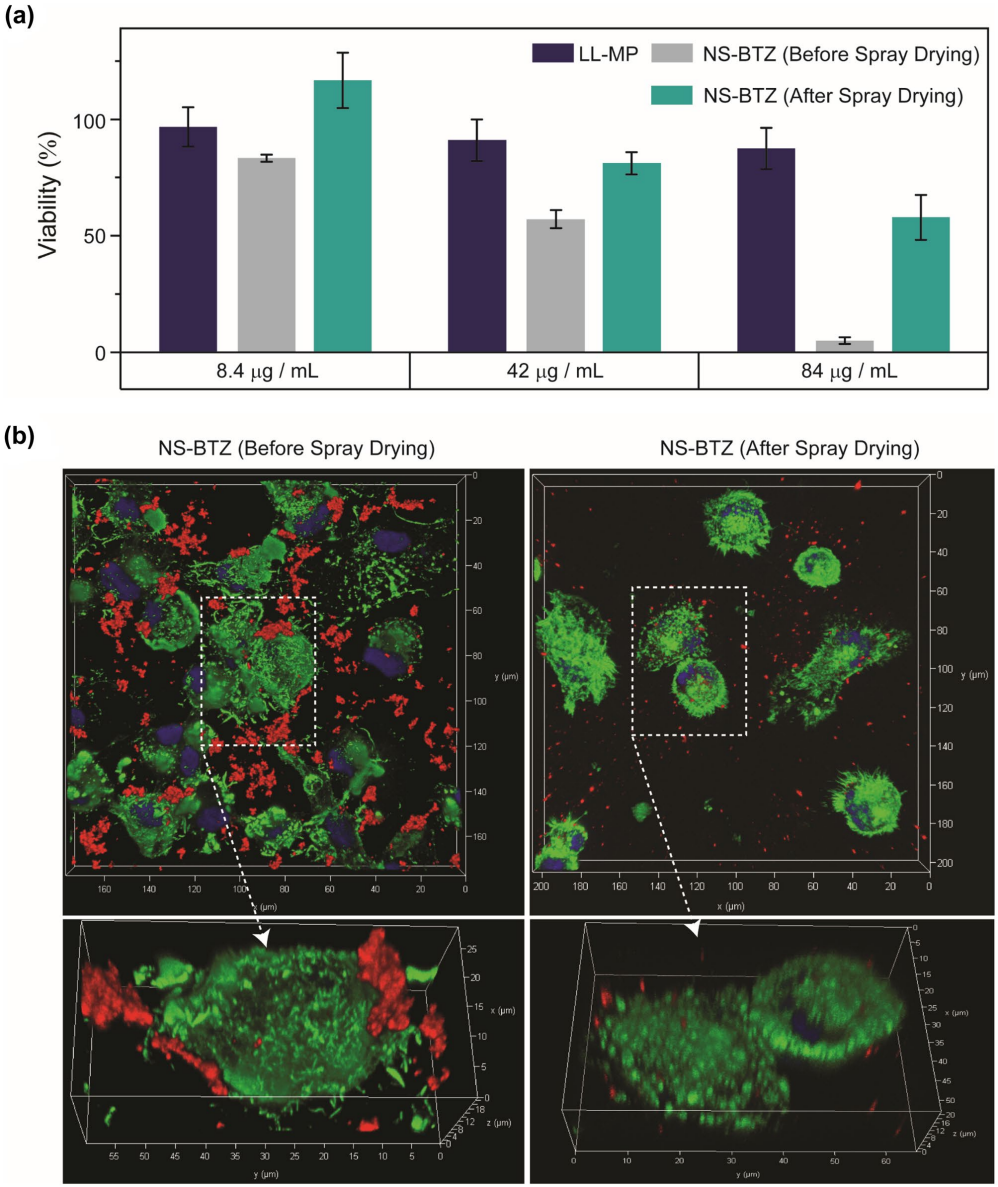
Cytotoxicity and cell uptake of benzothiazinone 043-loaded nanopharmaceuticals using a macrophage-like cell line. (**a**) Cytotoxicity measurement of NS-BTZ before and after spray drying (i.e., the dissolved NS-BTZ MP sample) using the MTT cell viability assay. (**b**) THP-1 uptake of NS-BTZ MPs analyzed by confocal microscopy. Nanosuspensions were labeled with lumogen red (red coloration), actin filaments were stained by Alexa Fluor 488-Phalloidin (green coloration), and nuclei were counterstained with DAPI (blue coloration). Frames of interest (top) have been magnified, tilted, and depicted three-dimensionally (bottom)

As an optimal drug formulation must be biocompatible, it needs to be non-toxic at least within the range of the expected therapeutic window. We here evaluated the cytotoxicity of the NS-BTZ samples before and after spray drying by measuring the metabolic activity of the differentiated macrophage-like cells by MTT assay. Of all the formulations employed in this study, we selected NS-BTZ for cell interaction studies because of their high fraction available for lung deposition, improved colloidal properties of the incorporated nanosystem after spray drying, and high API load. Alveolar macrophages, here represented by the PMA-differentiated human THP-1 cell line, are the predominant cell type for intracellular mycobacterial infections and were therefore selected as a model system [30, 33]. Remarkably, all spray-dried NS-BTZ particles showed consistently reduced cytotoxic effects on THP-1 cells compared to the non-spray dried material (Fig. 4a).

In addition, uptake by PMA-differentiated THP-1 cells of non-spray-dried vs. spray-dried NS-BTZ was investigated. Without spray drying, nanosuspensions showed aggregation as indicated by clusters of strong red fluorescence signals when offered to macrophages. In contrast, the spray-dried formulation showed a more homogeneously distributed and overall weaker red fluorescence signal of non-aggregating NPs when taken up by the reporter cells (Fig. 4b).

## Discussion

It may be assumed that spraying of aqueous dispersions leads to the formation of spherical droplets. In contact with hot air, the water will evaporate. During this event, the resulting dry particles might break or shrink because of the pressure exerted on the surface, eventually even leading to asymmetric or elongated structures. Interestingly, our results suggest that the SiNP-BTZ were well distributed inside the drying droplets, while all other NPs showed some tendency toward aggregation. This difference might be due to differing physicochemical characteristics of the NPs incorporated within the resulting dry powders. During spray drying, BTZ nanoformulations attained colloidal stability and a dispersed character, which might be enabled by the lactose molecules coating the NPs.

The physicochemical properties of NS-BTZ and SiNP-BTZ NPs obviously favor their homogeneous encapsulation within the MPs, which appeared very similar to plain LL-MPs. Nevertheless, FIB-SEM analysis revealed internal structures within the dry powder formulations of SiNP-BTZ particles, while such structures were absent from the NS-BTZ preparations. This might be due to the higher density and the defined nanostructure of the SiNP-BTZ, which allowed for its electron-microscopic detection. In contrast, the NS-BTZ nanosuspension of plain drug particles appeared to be either absorbed to the MPs’ inner lining or integrated within their overall structure, which in either case obviously obstructs their detectability. When encapsulating ZrNCs or Lip-TS-LVX NPs, the MP structures were significantly altered. Specifically, the hollow ZrNC influenced the aggregation considerably, and hollow spaces were observed inside the MPs. As mentioned in the literature [34], the morphology of the MPs may not be changed by the Péclet number but the colloidal properties of the nanosystem might have influenced the morphology of the MPs and the internal arrangements.

The aggregation of the soft Lip-TS-LVX nanosystem is in line with earlier findings that liposomal membrane fusion may occur upon their close spatial proximity inside MPs [25]. Despite the lactose surface coating of the liposomal formulation, the temperature and/or pressure exerted during the spray-drying process might have affected its integrity and fusogenicity. However, as these factors were kept constant for all spray-dried samples, we conclude that the observed MP characteristics are rather associated with the intrinsic chemical (composition) and physical (size) properties of the spray-dried nanomaterials. Furthermore, the agglomerates seen in the microscopic images of ZrNC was reduced when dissolving ZrNC-MPs in buffer. Thus, aggregation may be reversible and might be triggered upon reaching the pulmonary mucosa. All of these characteristics certainly influenced the median size of the MPs and the microscopic indications of agglomeration indeed correlated with the MPs’ sizes as determined by light scattering.

In a real-world scenario, the particles’ aerodynamic properties dictate their deposition in the lung [35]. In this regard, the experiments employing the NGI [32] revealed that the BTZ formulations had almost similar aerodynamic particle-size distributions and respirable fractions (fine particle mass: stages 1–5). As had been demonstrated by physicochemical analysis, these two particle variants exhibited similarity with LL-MPs and thus maintained similar aerodynamic properties. However, ZrNCs were very light, which apparently relates to their hollow structures. Indeed, the ZrNC NPs were purposefully prepared as hollow containers so as to enable the subsequent loading with various APIs as demonstrated previously [27]; this considerably influenced the density of the powder. As ZrNC MPs were dry and light (with a low density), they did not settle in any of the stages, which led to a significantly reduced respirable fraction. In contrast, MPs with a high density may have good aerodynamic properties and deep lung deposition. Interestingly, the observed changes in the shape of the Lip-TS-LVX MPs did not influence their aerodynamic properties, which corroborates the assumption that a spherical shape is not necessarily a precondition for optimal aerodynamic properties of a given MP.

Importantly, the FT-IR analysis revealed that non-encapsulated BTZ and LVX were crystalline in nature, which was not the case for spray-dried nanocarriers containing these drugs. This suggests that spray drying is also favorable to achieve amorphous structures [20, 36], which may facilitate the release of API-loaded NPs upon contact with the fluid of the alveolar lining. Indeed, efficient and rapid release of drug-loaded NPs at the site of infection is a prerequisite to address *M. tuberculosis* residing within infected alveolar macrophages as rapidly as possible.

Finally, both excipients — lactose [37, 38] and leucine [39] — are biocompatible molecules that are used as excipients in pulmonary applications. In fact, lactose is the only FDA-approved monosaccharide for lung applications (20, 37, 38). The excipients employed herein are thus expected to aid in dispersing some of the nanocarriers while at the same time minimizing possible toxic effects. The stronger aggregation of non-spray-dried BTZ-NS might be due to the direct interaction of certain cellular components with the surface of BTZ-NS that apparently are shielded in the presence of lactose and leucine.

## Conclusion

The physiochemical characteristics of the different nanocarriers employed in this study — including size, composition, zeta potential, density, and their colloidal properties — influenced the size and aerodynamic properties of the spray-dried powders. Interestingly, besides potentially enabling aerosol delivery to the deeper lung, the colloidal properties of certain nanoformulations were improved by the spray-drying process. In particular, the spray-dried BTZ nanosuspension showed better dispersibility and a different interaction with a macrophage-like cell line compared to the same nanomaterial before spray drying. While the colloidal properties were improved for such solid NPs post spray drying, soft low-density NPs tended to increase in size after spray drying due to some aggregation. Encouragingly, the relatively simple nanosuspension of plain API showed the most promising properties post spray drying. Such a product may be further evaluated in more complex biological models enabling to address *M. tuberculosis* infections more efficiently.

## Supplementary Information

Below is the link to the electronic supplementary material.

Supplementary file1 (DOCX 1840 KB)

## Data Availability

The authors confirm that the data supporting the findings of this study are available within the article and its supplementary materials. Raw data are available from the corresponding author, BL, upon reasonable request.

## Abbreviations

ANTI-TB: Project acronym for antibiotic nanocarriers for therapeutic inhalation against tuberculosis
API: Active pharmaceutical ingredient
BSA: Bovine serum albumin
BTZ: Benzothiazinone 043
DAPI: 4′,6-Diamidino-2-phenylindole
FIB-SEM: Focused ion beam scanning electron microscopy
FT-IR: Fourier transform infrared
HBSS: Hanks’ balanced salt solution
LVX: Levofloxacin
MPs: Microparticles
MTT: 3-(4,5-Dimethylthiazol-2-Yl)-2, 5-diphenyl tetrazolium bromide
NGI: Next generation impactor
NPs: Nanoparticles
PBS: Phosphate-buffered saline
PFA: Paraformaldehyde
PMA: Phorbol 12-myristate 13-acetate
RT: Room temperature
SDS: Sodium dodecyl sulfate
SEM: Scanning electron microscopy
XRD: X-ray diffraction
