# Supplementary material for: Spray-dried lactose-leucine microparticles for pulmonary delivery of antimycobacterial nanopharmaceuticals
Source: Drug Deliv Transl Res. 2021 Jun 8;11(4):1766–78. doi: 10.1007/s13346-021-01011-7 (PMC8236044; doi:10.1007/s13346-021-01011-7)
Supplement: Supplementary file 1 — Supplementary file1 (DOCX 1840 KB) [file 13346_2021_1011_MOESM1_ESM.docx]

Supporting Information for

Spray-dried Lactose-Leucine Microparticles for Pulmonary Delivery of

Antimycobacterial Nanopharmaceuticals

Durairaj Thiyagarajan1
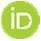
 0000-0003-2487-3415, Benedikt Huck1, 2
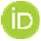
 0000-0003-3111-6009, Birgit Nothdurft^3^, Marcus Koch^3^, David Rudolph^4^, Mark Rutschmann^4^, Claus Feldmann^4^, Constantin

Hozsa5Δ
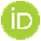
 0000-0001-7766-3644, Marcus Furch5ΔΔ, Karen F. W. Besecke5, Robert K. Gieseler5
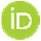


0000-0002-3064-8516, Brigitta Loretz1*
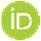
 0000-0003-0057-5181, Claus-Michael Lehr1, 2
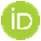
 0000-0002-5864-8462

^1^Helmholtz-Institute for Pharmaceutical Research Saarland (HIPS), Department of Drug

Delivery, Campus E8.1, 66123 Saarbrücken, Germany

^2^Department of Pharmacy, Saarland University, 66123 Saarbrücken, Germany

^3^INM – Leibniz Institute for New Materials, Campus D2 2, 66123 Saarbrücken, Germany

^4^Institute of Inorganic Chemistry, Karlsruhe Institute of Technology (KIT), Engesserstr. 15,

76131 Karlsruhe, Germany

^5^Rodos Biotarget GmbH, Feodor-Lynen-Str. 31, 30625 Hannover, Germany

^*^Corresponding author: Brigitta Loretz, Brigitta.Loretz@helmholtz-hips.de

^Δ^Current address: Siegfried AG Hameln, 31789 Hameln, Germany

^ΔΔ^Current address: Biolife Holding GmbH & Co. KG, 69126 Heidelberg, Germany

^^Department of Medicine, University Hospital Bochum, 44892 Bochum, Germany

Powder Characterization


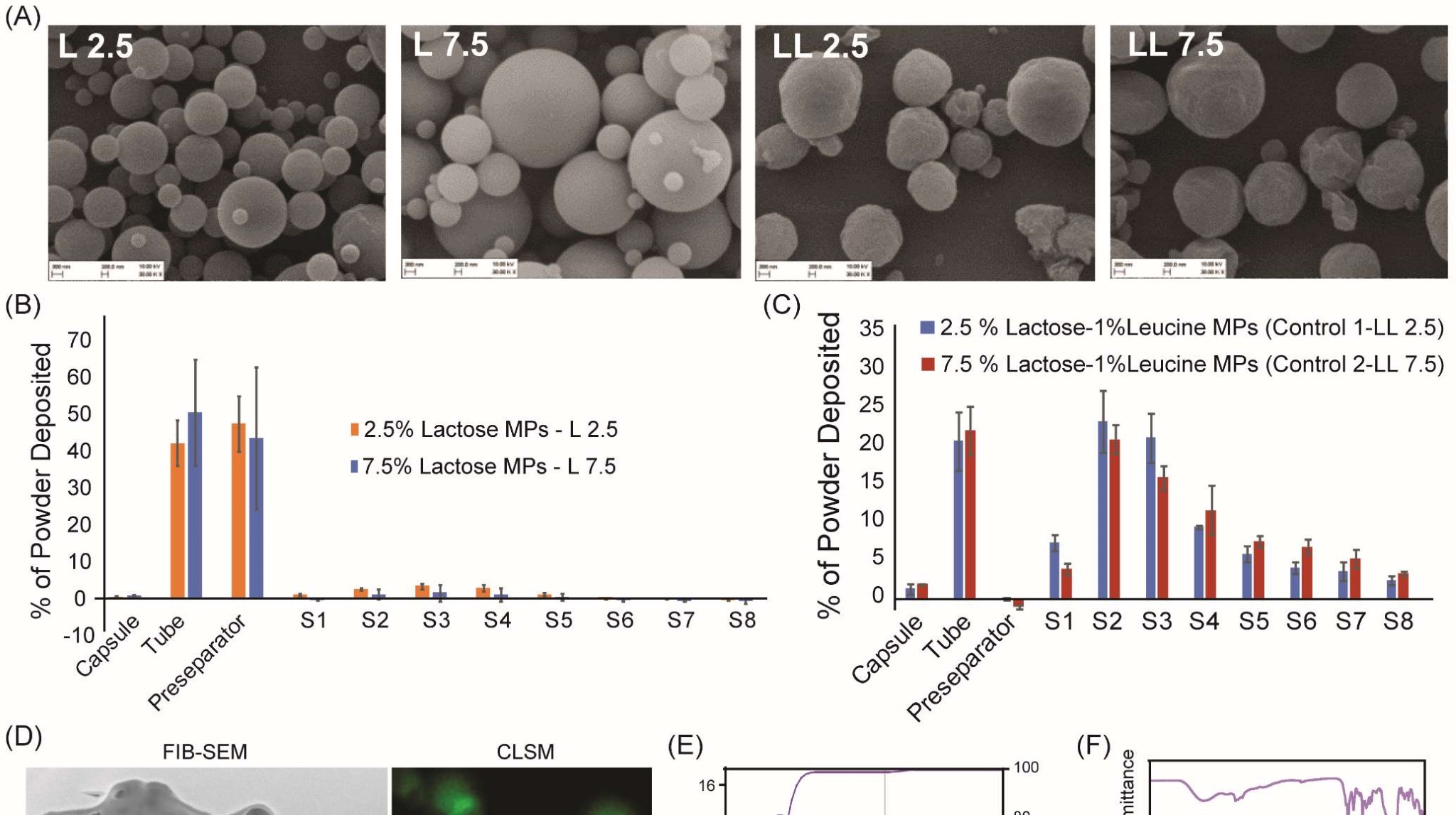

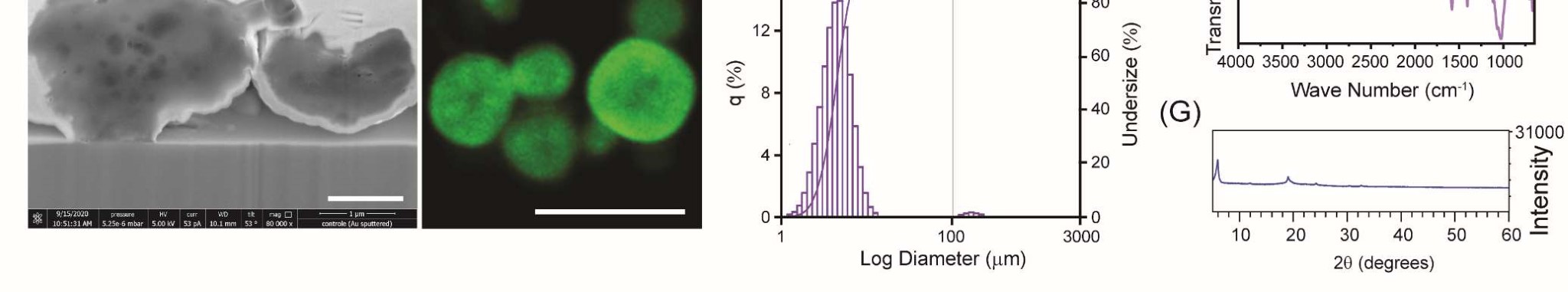


Figure 5 (A) Scanning Electron Microscopic analysis of plain lactose (L MPs) and

lactose/leucine microparticles (LL-MPs) as control sample. Characterization of (B) L MPs and C) LL MPs by next-generation impactor to check the aerodynamic properties. Further detailed characterization of selected 2.5% LL MPs by (D) FIB-SEM and confocal microscopy (E) static light scattering (F) FT-IR (G) XRD

.


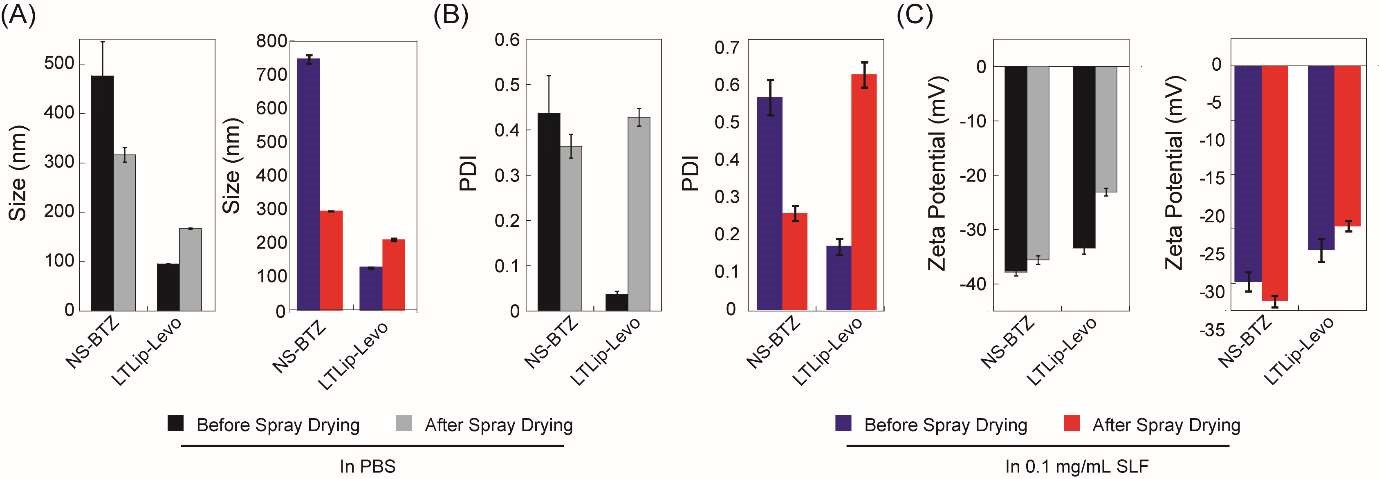


Figure 6 Assessment of colloidal properties of the nanocarriers in the 0.1 mg/mL Simulated Lung Fluid (SLF prepared as suggested in Reference 1) (A) size measurement by DLS and its (B) PDI values. (C) Zeta potential measurements. The results obtained with the PBS were replotted adjacent to the results obtained with 0.1 mg/mL SLF for the comparison.

API content quantification by HPLC


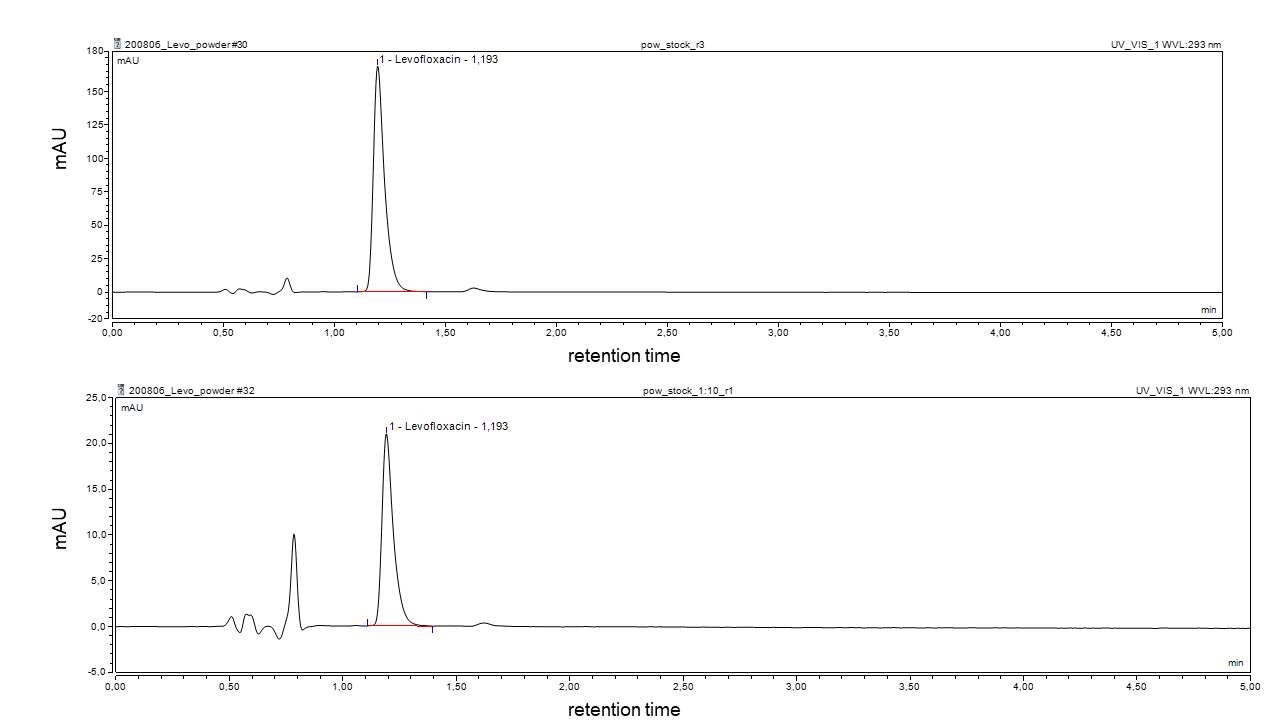


Lip-TS-LVX before spray drying

Lip-TS-LVX after spray drying

Figure 7. HPLC measurement of LFX in Lip-TS-LVX.


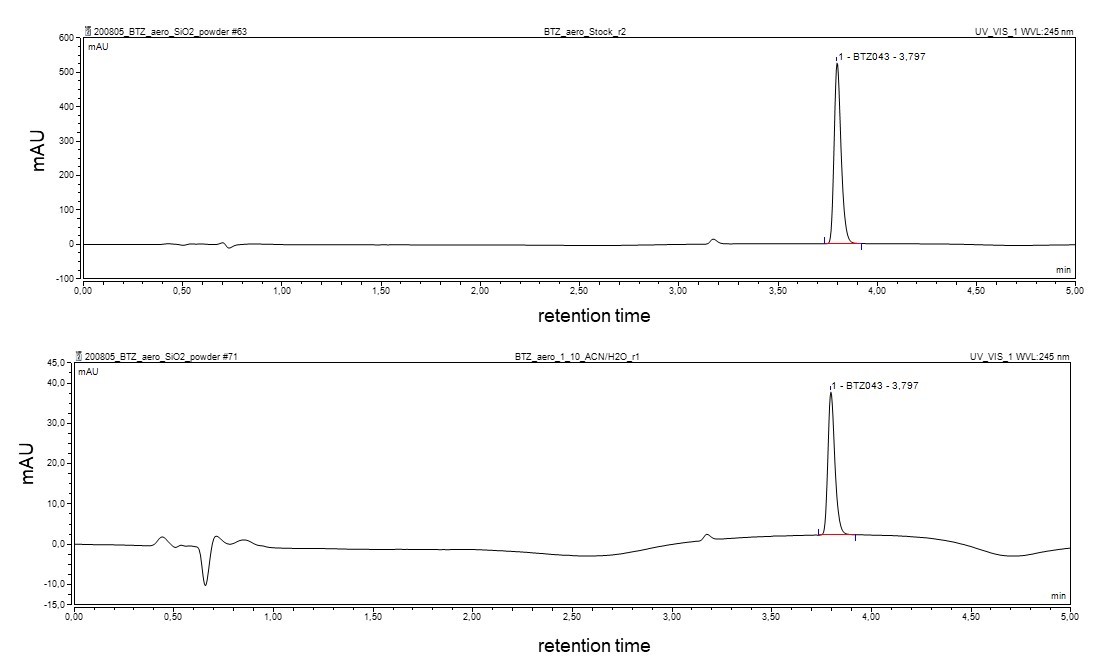


NS-BTZ before spray drying

NS-BTZ after spray drying

Figure 8. HPLC measurement of BTZ-043 in NS-BTZ.


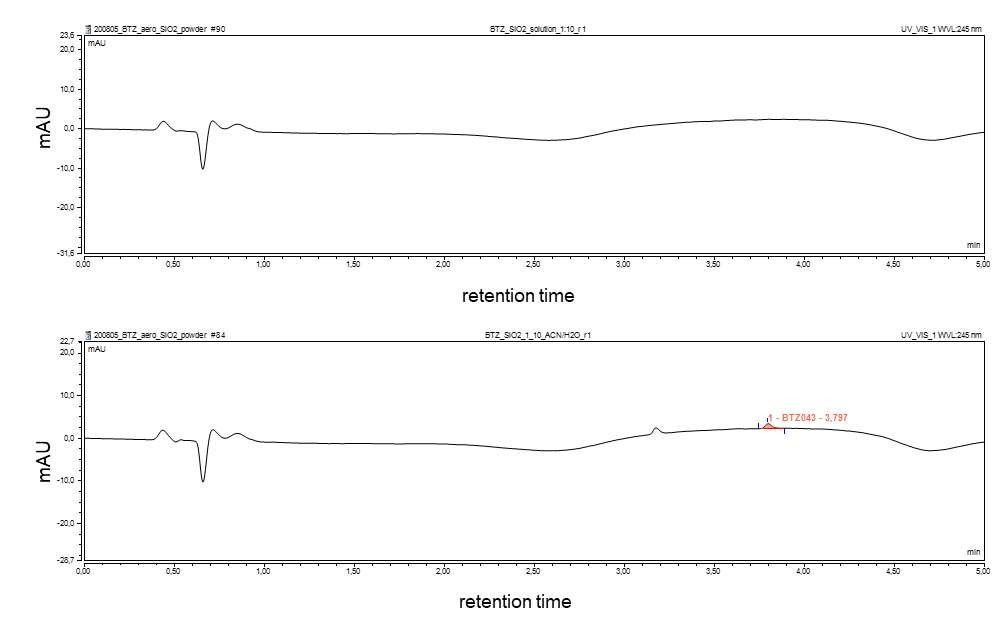


SiNP-BTZ before spray drying

SiNP-BTZ after spray drying

Figure 9. HPLC measurement of BTZ-043 in SiNP-BTZ.

References:

1. Kumar, A., Terakosolphan, W., Hassoun, M. et al. A Biocompatible Synthetic Lung Fluid Based on Human Respiratory Tract Lining Fluid Composition. Pharm

Res 34, 2454–2465 (2017). https://doi.org/10.1007/s11095-017-2169-4
